# Orthorexia nervosa and exercise addiction: distinct entities beyond restrictive and muscularity-oriented disordered eating behaviours?

**DOI:** 10.1186/s40337-026-01535-8

**Published:** 2026-01-25

**Authors:** Hanna Wachten, Jana Strahler

**Affiliations:** Department of Sport and Sport Science, Sportpsychology, Albert-Ludwigs-University Freiburg, Freiburg, Germany

**Keywords:** Disordered eating, Orthorexia nervosa, Exercise addiction, Clinical relevance, Impairment, Distress

## Abstract

**Background:**

Orthorexia Nervosa (OrNe) and Exercise Addiction (EA) are potentially dysfunctional variants of health-oriented behaviours, but their status as distinct mental disorders remains debated. OrNe is the obsessive preoccupation with ‘healthy’ eating, whereas EA is characterized by loss of control over exercise and prioritization over other life domains. Both commonly coincide with disordered eating, raising the question whether the clinical indicators are inherent to OrNe and EA or primarily reflect overlapping eating pathologies. This study examined whether OrNe and EA are distinct from restrictive and muscularity-oriented disordered eating by assessing their overlap and their unique links to psychological distress and psychosocial impairment.

**Methods:**

Within a cross-sectional web-based study, 384 participants (age = 31.5±11.5; 76.3% women) completed the Teruel Orthorexia Scale (TOS), Exercise Dependence Scale Revised (EDS-R), Eating Disorder Examination-Questionnaire (EDE-Q), Muscularity-Oriented Eating Test (MOET), Brief Symptom Inventory-18 (BSI-18), and Clinical Impairment Assessment Questionnaire (CIA).

**Results:**

Bivariate correlation analyses revealed strong overlaps of TOS-OrNe with both forms of disordered eating (EDE-Q: *r* = .635, MOET: *r* = .730), which were significantly more pronounced in women (EDE-Q: *r* = .676, MOET: *r* = .810) than men. EA was strongly correlated with MOET (*r* = .536), and weakly with EDE-Q (*r* = .242). Multiple regression and path analyses showed OrNe predicted psychosocial impairment and psychological distress both directly and mediated by EDE-Q, whereas EA was not uniquely linked to clinical indicators. Exploratory factor analysis further suggested strongly related latent constructs.

**Conclusion:**

Although OrNe was uniquely associated with psychological distress and impairment, its substantial overlap with restrictive and muscularity-oriented disordered eating challenges its validity as a distinct disorder. EA’s association with impairment appears largely explained by muscularity concerns, questioning its conceptualization as a behavioural addiction. These findings underscore the importance of considering muscularity-related motives and behaviours in both research and clinical assessment. Overall, OrNe and EA may reflect socioculturally shaped expressions of disordered eating rather than independent mental disorders.

**Supplementary Information:**

The online version contains supplementary material available at 10.1186/s40337-026-01535-8.

## Abstract (plain language summary)

Healthy eating and regular exercise usually support well-being, but for some people, focusing on these behaviours too intensely may cause problems in daily life. Two patterns that have received attention are Orthorexia Nervosa (OrNe) and Exercise Addiction (EA). OrNe involves being strongly focused on eating only ‘healthy’ foods, while EA involves a strong need to exercise, prioritizing it over work, family, or other daily activities. These behaviours often overlap with disordered eating, such as strict dieting or a focus on building muscle, so it is unclear whether any distress or daily-life problems are caused by OrNe and EA themselves or by disordered eating. Our study examined how much OrNe and EA are linked to restrictive and muscle-focused eating patterns and whether they contribute to mental health problems. We found that both OrNe and EA strongly overlap with these eating behaviours, meaning that people with these patterns are also likely concerned with thinness or muscularity. EA alone does not seem to cause mental health problems beyond those associated with disordered eating. Regardless of how these behaviours are classified in research, anyone who experiences distress or difficulties because of their eating or exercise habits may benefit from seeking professional support or treatment.

**Supplementary Information:**

The online version contains supplementary material available at 10.1186/s40337-026-01535-8.

## Background

While exercise and heathy eating are widely promoted for their physical and mental health benefits [1], their excessive pursuit may become pathological. Two controversial examples of such phenomena are *Orthorexia Nervosa* (OrNe) and *Exercise Addiction* (EA), which may reflect dysfunctional variants of health-oriented behaviours. According to the OrNe task force [2], OrNe is the strong preoccupation with “correct” or “healthy” eating. This involves spending an excessive amount of time planning, preparing, and consuming food, which impairs psychosocial functioning. Individuals with OrNe tend to hold strong beliefs about exaggerated or false health effects of foods. Therefore, violating one’s self-imposed dietary rules leads to psychological distress, such as guilt or anxiety [2–4]. As definitions of “healthy” eating vary between individuals and the range of permitted foods becomes increasingly restricted, malnutrition and underweight may occur subsequently [2]. Similarly, the loss of control over a health behaviour is present in the behavioural addiction Exercise Addiction. Specifically, EA refers to the prioritization of exercise in daily life at the expense of other activities, resulting in intrapsychic, social, or occupational conflicts. Exercise is used as means of mood regulation, while affected individuals tend to lack other adaptive emotional coping strategies and experience withdrawal symptoms when exercise is interrupted [5–7]. Over time, tolerance develops, leading to a need for increasingly frequent, intense, or prolonged exercise sessions to achieve effective mood regulation – often exceeding the originally intended amount. Attempts to reduce exercise behaviour often fail, even when adverse consequences (e.g., injuries) are present or when individuals are aware of maladaptive behaviour patterns [5, 6]. Despite growing attention, it remains a matter of debate whether OrNe and EA qualify as distinct mental disorders.

One major concern is the limited empirical evidence of their clinical relevance beyond case studies [8, 9]. In this regard, most studies found associations with higher psychological distress, such as anxiety, stress, or depression [e.g. 10–15]. For instance, EA was associated with higher stress and depressiveness among athletes in general, while injured athletes were at higher risk for depression when also being at risk for EA [16]. Some studies also take psychosocial impairment into account. EA has been linked to reduced social behaviour in a large student sample [17] and lower general functioning among marathon runners [11]. Research on the clinical relevance of OrNe has been hampered by the widespread use of the ORTO-15 and its versions, which primarily capture interest in healthy eating rather than pathological manifestations [18, 19]. The Teruel Orthorexia Scale [20] addresses this limitation by distinguishing between healthy Orthorexia and Orthorexia nervosa – two constructs that, as expected, differ in their associations with depression, anxiety, and stress [10, 14, 21]. However, it remains unclear whether psychological distress and functional impairment represent risk factors, clinical indicators, or whether they primarily reflect underlying or co-occurring conditions.

In particular, the frequent overlap with symptoms of eating disorders raises the question of whether OrNe and EA represent distinct clinical entities or merely variants within the broader spectrum of disordered eating. Although there is consensus that weight control is not a primary or explicit concern in OrNe [2], evidence suggests that it may, in fact, be a key motive for food choice rather than health-related motives [22, 23]. According to a meta-analysis, symptoms of disordered and orthorexic eating behaviour overlap moderately with *r* = 0.36 [24]. Specifically, OrNe seems to be related to restrictive disordered eating, dieting behaviours, and drive for thinness, but not purging behaviour such as excessive exercise [25]. The latter is used instrumentally to control both one’s weight and mood in eating disorders [26, 27], and contributes to the overlap of EA with disordered eating [28]. EA is 3.5 times more likely to co-occur with disordered eating than without [29], in which case it was referred to as secondary EA [30]. Recent work, however, suggests that secondary EA reflects instrumental exercise within disordered eating and should not be classified as a behavioural addiction, as continued use of the term may lead to conceptual confounding [31, 32]. Indeed, it is possible that the moderate correlations of EA with symptoms of disordered eating, drive for thinness, and weight, shape, or body image concerns [33–35] stem from confounding with instrumental exercise. Moreover, other forms of eating pathology should be taken into account when examining its overlap with OrNe and EA.

Traditional definitions of disordered eating – focused on dietary restrained and drive for thinness – may not fully capture male expressions of eating pathology. In men, dysfunctional eating behaviours often revolve around a drive for muscularity to align with the idealized large shape [36, 37]. Such *muscularity-oriented disordered eating behaviours* – including high protein intake, rigid meal timing, excessive exercise, supplement or steroid use – are increasingly recognized as components of eating pathology in both men and women [38–42]. Amid changing social norms, e.g. in social media [43], the ideal female body has shifted from a solely thin ideal toward an athletic, toned, and moderately muscular appearance [44, 45]. To date, evidence on the role of muscularity in EA remains limited. Associations of EA with muscularity-oriented disordered eating was found in a student sample [46], and muscularity concerns in male bodybuilders and weight lifting athletes [47–49]. Two recent network analyses of non-clinical samples have underscored the relevance of muscularity and orthorexic concerns within disordered eating. In men, muscularity-oriented disordered eating prominently featured depressive symptoms following missed training sessions, resembling withdrawal symptoms in EA, and is closely associated with rumination on healthy eating [50]. In women, rumination about healthy eating related broadly to various disordered eating symptoms, including muscularity concerns, fear and guilt linked to weight gain [40]. Given that nutrition is commonly perceived as “healthy” when low in fat and carbohydrates but high in protein [51], “healthy eating” may be conflated with muscularity-oriented eating – potentially obscuring the actual motivation behind such behaviours. In line, perceptions of “health” varied between individuals at risk for OrNe, with some considering a toned physique as an essential component of health [52]. An recent study further examined orthorexic tendencies using both explicit and implicit measures of body image attitudes and distortions [53]. Implicit distortions were assessed via a reverse-correlation approach, in which participants generated representations of their actual, perceived, and ideal body, and independent raters evaluated deviations from reality, particularly regarding muscularity. OrNe was not only linked to explicit weight and health-related attitudes but also to implicit biases towards muscular body ideals and distortions. Consistent with these findings, a five-case qualitative study examined individuals with varying levels of orthorexic symptoms [3]. Three of these cases showed an emphasis on high protein intake, avoidance of wasting calories, fear of weight gain, athletic goal pursuit, or disordered eating. Although all cases reported signs of either illness anxiety or health optimalization, levels of impairment and psychological distress varied. Only one case exhibited overvalued – possibly delusional – beliefs about the effects of nutrition.

Overall, it remains unclear whether psychological distress or impairment observed in OrNe and EA are better explained by underlying restrictive or muscularity-oriented eating pathology. Few studies statistically controlled for restrictive disordered eating when investigating their clinical relevance. In a sample of fitness center attendees, EA was found to be directly linked to elevated psychological distress, even when controlling for eating disorder risk [7]. Previous research has shown mixed findings regarding OrNe, with some studies reporting inconsistent associations with impairment [54, 55] and no links with well-being [56]. To our knowledge, no study has accounted for muscularity-oriented disordered eating. The aim of this study is twofold: First, to examine the extent to which OrNe and EA overlap with restrictive and muscularity-oriented disordered eating, and whether this association is moderated by gender. Second, to investigate whether OrNe and EA uniquely contribute to psychological distress and psychosocial impairment, beyond the effects of disordered eating symptoms. To address these aims, the following hypotheses were formulated: OrNe and EA demonstrate gender-specific patterns of association. While both are expected to correlate with disordered eating, stronger associations with muscularity-oriented behaviours are anticipated in men, and with restrictive behaviours in women. Furthermore, following the adjustment for restrictive and muscularity-oriented disordered eating, it is hypothesized that OrNe and EA will demonstrate distinctive associations with psychological distress and functional impairment.

## Methods

### Participants and data collection

Participants were recruited online (www.soscisurvey.de) using convenience sampling strategies, i.e. flyers were distributed in public places and social media. The study was advertised as “exercise, nutrition and health study” in German language. The inclusion criteria were age of majority (≥ 18 years) and that participants were not currently undergoing inpatient psychiatric treatment. All participants gave informed consent and were rewarded with the opportunity to enter a prize draw for ten 20€ vouchers. The data was combined from two cross-sectional survey rounds (first: *N* = 224, second: *N* = 160), with no missing data for any variables, as the survey system required participants to complete all items. Since analyses were performed for women and men, we excluded four participants who indicated their gender as *diverse*, resulting in a total sample size of *N* = 384. This study was preregistered on PsychArchives [57]. In deviation from the preregistration, the TOS was used instead of the Düsseldorf Orthorexia Scale (DOS) to assess OrNe, as the DOS’s may conflate pathological and non-pathological healthy eating behaviours [24, 54]. In addition, the preregistration indicated the use of SEM and measurement invariance to examine gender differences; however, regression models and an exploratively path analysis were conducted instead.

### Measures

Participants provided sociodemographic information, including age, gender (woman, man, diverse), educational and professional qualifications, dietary style (omnivorous, vegetarian, vegan, other), and whether they restrict their diet for medical or religious/cultural reasons. They also reported current or past engagement in outpatient psychotherapy.

The Orthorexia Nervosa scale of the Teruel Orthorexia Scale (TOS-OrNe; [20]) assessed orthorexic eating behaviour. Eight items are rated on 4-point Likert scales (e.g., “If, at some point, I eat something that I consider unhealthy, I punish myself for it”), with response options ranging from “0 – completely disagree” to “3 – completely agree”. Higher sum scores reflect elevated OrNe levels (range: 0–24), while cut-off values are not provided. The second scale *Healthy Orthorexia*, which assesses the non-pathological interest in healthy eating on nine items, was not analysed in this study. Different associations of both subscales with anxiety, stress, depression, well-being, and affect indicate good construct validity [14, 58]. Cronbach’s α was excellent with 0.89, 95%-CI [0.87, 0.90] in this sample.

The Exercise Dependence Scale Revised (EDS-R; [59,60]) measured EA, i.e. its symptoms tolerance, withdrawal, intention effects, lack of control, time, reduction in other activities, and continuance. Participants respond to 21 items using 6-point Likert scales from “1 – never“ to “6 – always“. Higher sum scores indicate higher EA levels (range: 21–126), and item responses can be used to categorize participants into three groups, i.e. nondependent–asymptomatic, nondependent–symptomatic, and at risk for EA. Factor validity and reliability of both the original and German version was acceptable to good [59, 60]. The EDS-R seems to be a valid tool to assess EA across competition levels of athletes, with the cut-off identifying athletes, who are less likely to adhere to medical advice to stop exercising [61]. Compared to other instruments, the EDS-R produces lower overlaps with disordered eating (OR = 2.4; [29]). Cronbach’s α was excellent with 0.95, 95%-CI [0.94, 0.95] in this sample.

Muscularity-oriented disordered eating behaviours were assessed by the Muscularity-Oriented Eating Test (MOET; [42]). Fifteen items are rated on 5-point Likert scales (e.g., “I tracked the macronutrients of everything I ate”), with response options ranging from “0 – never true” to “4 – always true”. Higher mean scores (range: 0–4) indicate higher levels of muscularity oriented pathological eating behaviours. We translated and validated a German version of the MOET with a sample size of *N* = 525 (mean age: 31.16 ± 11.21, 68.6% women). The confirmatory factor analyses suggest acceptable factorial validity with a unidimensional structure (CFI = 0.968, SRMR = 0.068, RMSEA = 0.076 90% CI [0.063; 0.089]). Metric measurement invariance across gender was supported, while scalar invariance was not, indicating item threshold differences between men and women. Construct validity was supported by strong correlations with eating pathology and muscle dysmorphia measures, and moderate associations with self-esteem and psychological distress. This data is not currently published, but is available as part of the study documentation on reasonable request. Cronbach’s α was excellent (α = 0.91, 95%-CI[0.90, 0.92]) in this sample.

Restrictive disordered eating was measured by the Eating Disorder Examination Questionnaire (EDE-Q; [62,63]). Twenty-two items assess the severity or frequency of symptoms of eating disorders within the past 28 days, with response options regarding their severity or frequency on 7-point Likert scales from “0 – no days/never/not at all” to “6 – every day/always/very much”. Four subscales (Restraint, Eating Concern, Weight Concern, Shape Concern) and a global mean score (range: 0–6) can be computed. Higher mean scores indicate higher levels of eating disorder symptomatology. In addition, participants report the frequency of binge eating and compensatory behaviour on six open items. The EDE-Q is a well-established and valid instrument in both research and health care, with a high accuracy of the global score when discriminating participants with eating disorders from those without [64]. Cronbach’s α was excellent with 0.96, 95%-CI [0.95, 0.96] in this sample.

Psychological distress was assessed by the global severity index (GSI) of the Brief Symptom Inventory 18 (BSI-18; [65,66]). Participants respond to eighteen items using 5-point Likert scales from “0 – Not at all” to “4 – Extremely”, which measure anxiety, depression and somatization with six items each. The GSI is calculated as sum scale of all items, with higher scores reflecting higher symptom levels. Reliability and item discrimination of the German version was acceptable to good within student, healthy, and out-patient samples [67]. Cronbach’s α was excellent with 0.92, 95%-CI [0.90, 0.93] in this sample.

The Clinical Impairment Assessment Questionnaire (CIA; [68]) measured psychosocial impairment secondary to eating disorders, i.e. “how your eating habits, exercising or feelings about your eating, shape or weight have affected your life over the past four weeks (28 days)”. The 16 items probe impairment in domains mood, self-perception, cognitive functioning, interpersonal functioning, and work performances, with 7-point Likert scales from “0 – Not at all” to “3 – A lot”. The CIA was developed to be used immediately after the EDE-Q. Employing the CIA was suitable for our aims since its instructions ask participants to indicate how their eating habits, exercise or feelings about their eating, shape or weight have affected their life. Higher sum scores (range: 0–48) reflect higher severity, with a cut-off ≥ 16 indicating clinically significant impairment by eating disorder symptoms. The cut-off showed high sensitivity of 76% and specificity of 86% when differentiating between general and clinical samples. Moreover, the CIA was sensitive to treatment change and showed good construct and discriminant validity [69]. Cronbach’s α was excellent with 0.95, 95%-CI [0.95, 0.96] in this sample.

### Statistical analysis

All statistical analyses were conducted in R v4.4.2 [70] using the packages jtools [71] for regression analyses, mvnormalTest [72] for testing multivariate normality, and lavaan [73] for the path analysis. Descriptive statistics were computed as frequencies (*N*, %) for categorical variables, and as means, standard deviations (*M* ± *SD*), minima, and maxima [*Min*, *Max*] for continuous variables. To explore whether gender could act as a possible covariate, gender effects were then tested using Χ²-tests or Fisher’s exact tests, and *t*-tests or Welch’s *t*-tests when variances were not equal.

The hypotheses of moderated associations were tested using gender-stratified Pearson correlations of both MOET and EDE-Q with TOS-OrNe and EDS-R, and subsequently comparing those correlations between women and men using *z*-tests. Moreover, the correlation matrix of all variables of interest for the total sample and stratified by gender was exploratively reported to gain additional insight into the relationships of the data. To test the hypotheses of unique associations with indicators of clinical relevance, hierarchical and standardized regression analyses with the dependent variables GSI and CIA were conducted. Next to EDE-Q and MOET, gender was included as covariate in all regression analyses, since women and men differed significantly on all variables of interest except for TOS-OrNe and MOET. In Model 2, either TOS-OrNe or EDS-R were included in the model to test their incremental contribution. Due to heteroskedasticity and non-normality of residuals, HC4 robust standard errors were used [74, 75]. No influential outliers were detected (Cook’s distances < 0.5) and multicollinearity was ruled out (Variance Inflation Factor < 5).

To more thoroughly examine construct overlap, we exploratorily performed a path analysis including all variables of interest, employing maximum likelihood estimation and robust standard errors with 1000 bootstrap draws. According to the rule of thumb with a ratio of 20:1 for sample size and parameters, our sample size *N* = 384 > 340 with 17 parameter estimates was sufficiently large [76]. Acceptable model fit was indicated by the combination of Comparative Fit Index (CFI) ≥ 0.95 and Standardized Root Mean Squared Residual (SRMR) ≤ 0.08, since the usage of Root Mean Square Error of Approximation is not recommended with low degrees of freedom [77, 78]. Furthermore, an exploratory factor analysis (EFA) on TOS-OrNe, EDS-R, EDE-Q, and MOET was conducted to explore potential cross-loadings. Given the ordinal response scales, polychoric correlations with weighted least squares estimation were used [79]. Sampling adequacy was low but acceptable (Kaiser-Meyer-Olkin Criterion = 0.57), and Bartlett’s test indicated sufficient inter-item correlations, χ²(2145) = 84,721.82, *p* <.001. The number of factors was determined via parallel analysis and Velicer’s Minimum-Average-Partial test with polychoric correlations [80, 81]. Promax rotation was applied and loadings ≥ 0.264 were considered sufficient to be practically useful, following the recommendations for sample size-based cut-offs [82].

## Results

### Sample description

The sample (*N* = 384, mean age: 31.5 ± 11.5) consisted predominantly of women (76.3%), participants with higher education entrance qualifications (91.9%), and individuals following omnivorous (45.3%) or vegetarian (31.3%) diets (see Table 1). Most participants (70.0%) had never received psychotherapy; 12.0% were currently in outpatient treatment and 20.6% had received treatment in the past. Women were significantly younger (*t*(382) = 2.142, *p* =.033), had more frequent vegetarian dietary styles (χ² (3) = 11.662, *p* =.008), and were more likely to restrict their diet for medical (χ² (1) = 5.895, *p* =.015) but not religious or cultural reasons (*p* =.199) than men. Women and men did not differ in school qualification (*p* =.917) or professional qualification (*p* =.196). While women reported higher levels of restrictive disordered eating (EDE-Q: *t*(187.10) = − 3.636, *p* <.001), psychological distress (BSI-18: *t*(382) = − 2.059, *p* =.040), and psychosocial impairment (CIA: *t*(179.38) = − 2.460, *p* =.015), they scored lower on EDS-R (*t*(382) = 3.879, *p* <.001). No gender differences were found for TOS-OrNe (*t*(382) = − 1.112, *p* =.267) or muscularity-oriented disordered eating (MOET: *t*(382) = 1.752, *p* =.081).

**Table 1:** Sample description using descriptive statistics *M* ± *SD* [*Min*, *Max*] or *N* (%) of the total sample (*N* = 384) and stratified by gender

	All	Women	Men
Gender			
Women	293 (76.3)	293 (100)	-
Men	91 (23.7)	-	91 (100)
Age^a^	31.47 ± 11.50 [18; 69]	30.77 ± 11.10 [18, 68]	33.71 ± 12.52 [18, 69]
School qualification^b^			
None	–	–	–
School-leaving certificate	4 (1.0)	2 (0.7)	1 (1.1)
Intermediate school-leaving certificate	27 (7.0)	3 (1.0)	1 (1.1)
Higher education entrance qualifications	353 (91.9)	270 (92.2)	83 (91.2)
Professional qualification^b^			
None/Still in training	121 (41.3)	108 (36.9)	23 (25.3)
Apprenticeship/Vocational training	35 (9.1)	28 (9.6)	7 (7.7)
Advanced Vocational Qualifications	23 (6.0)	15 (5.1)	8 (8.8)
Higher Education	190 (49.5)	138 (47.1)	52 (57.1)
Other	5 (1.3)	4 (1.4)	1 (1.1)
Dietary style^a^			
Omnivorous	174 (45.3)	121 (41.3)	53 (58.2)
Vegetarian	120 (31.3)	104 (35.5)	16 (17.6)
Vegan	27 (7.0)	21 (7.2)	6 (6.6)
Other	63 (16.4)	47 (16.0)	16 (17.6)
Dietary restriction due to			
Medical reasons^a^	58 (15.1)	52 (17.8)	6 (6.6)
Religious or cultural reasons^b^	13 (3.4)	8 (2.7)	5 (5.5)
TOS-OrNe^b^	3.02 ± 4.07 [0, 23]	3.15 ± 4.23 [0, 23]	2.60 ± 3.47 [0, 18]
EDS-R^a^	49.16 ± 19.99 [21, 115]	46.99 ± 19.35 [21, 108]	56.13 ± 20.52 [21, 115]
EDE-Q^a^ ≥ 2.3	1.29 ± 1.24 [0, 5.88] 72 (18.8)	1.41 ± 1.28 [0, 5.45] 64 (21.8)	0.93 ± 1.01 [0, 5.88] 8 (8.8)
MOET^b^	0.65 ± 0.66 [0, 3.40]	0.61 ± 0.64 [0, 3.07]	0.75 ± 0.72 [0, 3.40]
BSI-18^a^	9.92 ± 10.00 [0, 69]	10.51 ± 10.61 [0, 69]	8.04 ± 7.48 [0, 33]
CIA^a^ ≥ 16	6.43 ± 8.56 [0, 48] 49 (12.8)	6.97 ± 8.86 [0, 48] 42 (14.3)	4.69 ± 7.31 [0, 46] 7 (7.7)

CIA: Clinical Impairment Assessment; EDE-Q: Eating Disorders Examination Questionnaire; EDS-R: Exercise Dependence Scale; BSI-18: Global Severity Index of the Brief Symptom Inventory 18; MOET: Muscularity-Oriented Eating Test; TOS-OrNe: Orthorexia nervosa subscale of the Teruel Orthorexia Scale

^a^Sig. gender effect

^b^Not sig. gender effect

### Bivariate correlations

**Fig. 1:**
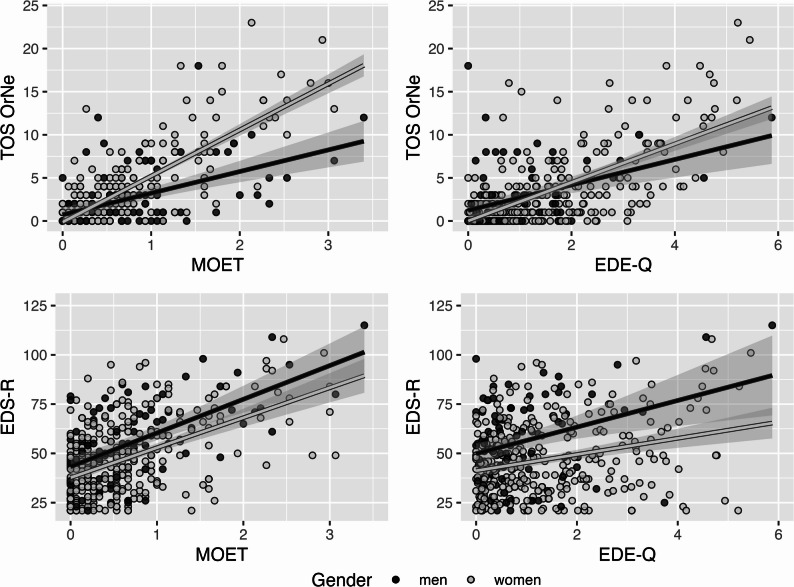
Scatter plots of orthorexic behaviours (TOS-OrNe) and exercise addiction (EDS-R) with restrictive (EDE-Q) and muscularity-oriented (MOET) disordered eating for women (N = 293) and men (*N* = 91). TOS-OrNe: Orthorexia nervosa subscale of the Teruel Orthorexia Scale; EDS-R: Exercise Dependence Scale; EDE-Q: Eating Disorders Examination Questionnaire; MOET: Muscularity-Oriented Eating Test

Across the total sample, TOS-OrNe showed strong correlations with the EDE-Q (*r* = 0.635, 95%-CI [0.571, 0.691], *p* <.001), and MOET (*r* = 0.730, 95%-CI [0.680, 0.774], *p* <.001). In contrast, the EDS-R correlated only slightly with the EDE-Q (*r* = 0.242, 95%-CI [0.146, 0.335], *p* <.001), but strongly with MOET (*r* = 0.536, 95%-CI [0.460, 0.603], *p* <.001). When examining gender-specific patterns, meaningful differences emerged for OrNe’s overlaps (see Fig. 1). Among women, TOS-OrNe was more strongly correlated with both EDE-Q (*r*_*w*_ = 0.676, 95%-CI [0.608, 0.734], *p* <.001), and MOET (*r*_*w*_ = 0.810, 95%-CI [0.766, 0.846], *p* <.001) than among men (*r*_*m*_ = 0.431, 95%-CI [0.247, 0.585], *p* <.001 and *r*_*m*_ = 0.524, 95%-CI [0.356, 0.659], *p* <.001, respectively). These differences were statistically significant (EDE-Q: *z* = 2.963, *p* =.002; MOET: *z* = 4.480, *p* <.001). In contrast, the correlations between EDS-R and EDE-Q (*r*_*w*_ = 0.272, 95%-CI [0.163, 0.375], *p* <.001 vs. *r*_*m*_ = 0.334, 95%-CI [0.137, 0.505], *p* =.001, with *z* = − 0.561, *p* =.287) or MOET (*r*_*w*_ = 0.504, 95%-CI [0.413, 0.584], *p* <.001 vs. *r*_*m*_ = 0.604, 95%-CI [0.454, 0.720], *p* <.001, with *z* = − 1.189, *p* =.117) did not significantly differ by gender.

Regarding indicators of clinical relevance among the total sample, TOS-OrNe was moderately and strongly associated with BSI-18 (*r* = 0.448, 95%-CI [0.364, 0.524], *p* <.001) and CIA (*r* = 0.667, 95%-CI [0.607, 0.719], *p* <.001), respectively. The EDS-R, however, was only slightly correlated with BSI-18 (*r* = 0.105, 95%-CI [0.005, 0.203], *p* =.041) and CIA (*r* = 0.228, 95%-CI [0.193, 0.377], *p* <.001). Notably, the BSI-18 was significantly correlated with TOS-OrNe (*r*_*w*_ = 0.510, 95%-CI [0.420, 0.590], *p* <.001; *r*_*m*_ = 0.083, 95%-CI [− 0.125, 0.284], *p* =.433) and EDS-R (*r*_*w*_ = 0.141, 95%-CI [0.027, 0.252], *p* =.015; *r*_*m*_ = 0.079, 95%-CI [− 0.129, 0.281], *p* =.456) in women, but not in men. The CIA, on the other hand, showed significant correlations with TOS-OrNe (*r*_*w*_ = 0.696, 95%-CI [0.632, 0.751], *p* <.001; *r*_*m*_ = 0.522, 95%-CI [0.355, 0.658], *p* <.001) and EDS-R (*r*_*w*_ = 0.276, 95%-CI [0.167, 0.379], *p* <.001; *r*_*m*_ = 0.479, 95%-CI [0.303, 0.624], *p* <.001) in both genders. See Table S1 (Supplementary Material) for correlations among all study variables, reported for the total sample and by gender.

### Regression and path analyses

The incremental contribution of TOS-OrNe and EDS-R to the clinical relevance indicators CIA and BSI-18 was examined using hierarchical regression analyses (see Table 2 for standardized coefficients). In Model 1, the EDE-Q, MOET, and gender together accounted for a substantial proportion of the variance in CIA scores (*R²* = 0.641, Adj. *R²* = 0.637, *F*(3,380) = 226.315, *p* <.001). The addition of TOS-OrNe significantly improved the model (Δ*R²* = 0.018, *F*(1, 379) = 20.166, *p* <.001), whereas the inclusion of EDS-R did not yield a significant change (Δ*R²* = 0.000, *F*(1, 379) = 0.053, *p*=.818). Furthermore, a small to moderate proportion of variance in BSI-18 scores was explained by the EDE-Q, MOET, and gender combined in Model 1 (*R²* = 0.283, Adj. *R²* = 0.277, *F*(3,380) = 49.974, *p* <.001). As observed for the CIA model, adding TOS-OrNe significantly increased explained variance of the BSI-18 scores (Δ*R²* = 0.028, *F*(1, 379) = 15.606, *p* <.001), while the EDS-R did not (Δ*R²* = 0.001, *F*(1, 379) = 0.433, *p* =.511).

**Table 2:** Standardized regression coefficients of the four hierarchical regression models explaining psychosocial impairment (CIA) or psychological distress (BSI-18) for the total sample (*N* = 384), with adding TOS-OrNe or EAI-R in the second model

Predictors	CIA			BSI-18		
	β [95% CI]	t	*p*	β [95% CI]	t	*p*
Model 1						
EDE-Q	**0.606 [0.478; 0.733]**	9.342	< .001	**0.521 [0.348; 0.694]**	5.934	< .001
MOET	**0.267 [0.119; 0.414]**	3.546	< .001	0.012 [− 0.138; 0.161]	0.154	.878
Gender	0.090 [− 0.054; 0.234]	1.233	.218	0.050 [− 0.135; 0.234]	0.528	.598
Model 2: TOS-OrNe						
TOS-OrNe	**0.221 [0.066; 0.357]**	2.851	.005	**0.264 [0.080; 0.449]**	2.813	.005
EDE-Q	**0.546 [0.401; 0.691]**	7.391	< .001	**0.446 [0.268; 0.625]**	4.927	< .001
MOET	0.147 [− 0.001; 0.295]	1.953	.052	− 0.137 [− 0.303; 0.028]	− 1.629	.104
Gender	0.060 [0.081; 0.200]	0.835	.404	0.011 [−0.176; 0.199]	0.120	.904
Model 2: EDS-R						
EDS-R	0.009 [− 0.093; 0.110]	0.166	.868	− 0.034 [− 0.140; 0.071]	− 0.640	.552
EDE-Q	**0.606 [0.476; 0.737]**	9.135	< .001	**0.518 [0.345; 0.691]**	5.875	< .001
MOET	**0.262 [0.084; 0.439]**	2.904	.004	0.032 [− 0.129; 0.192]	0.388	.698
Gender	0.093 [− 0.045; 0.231]	1.324	.186	0.039 [− 0.144; 0.222]	0.421	.674

Bold values indicate significant regression coefficients

CIA: Clinical Impairment Assessment; EDE-Q: Eating Disorders Examination Questionnaire; EDS-R: Exercise Dependence Scale; BSI-18: Global Severity Index of the Brief Symptom Inventory 18; MOET: Muscularity-Oriented Eating Test; TOS-OrNe: Orthorexia nervosa subscale of the Teruel Orthorexia Scale

**Fig. 2:**
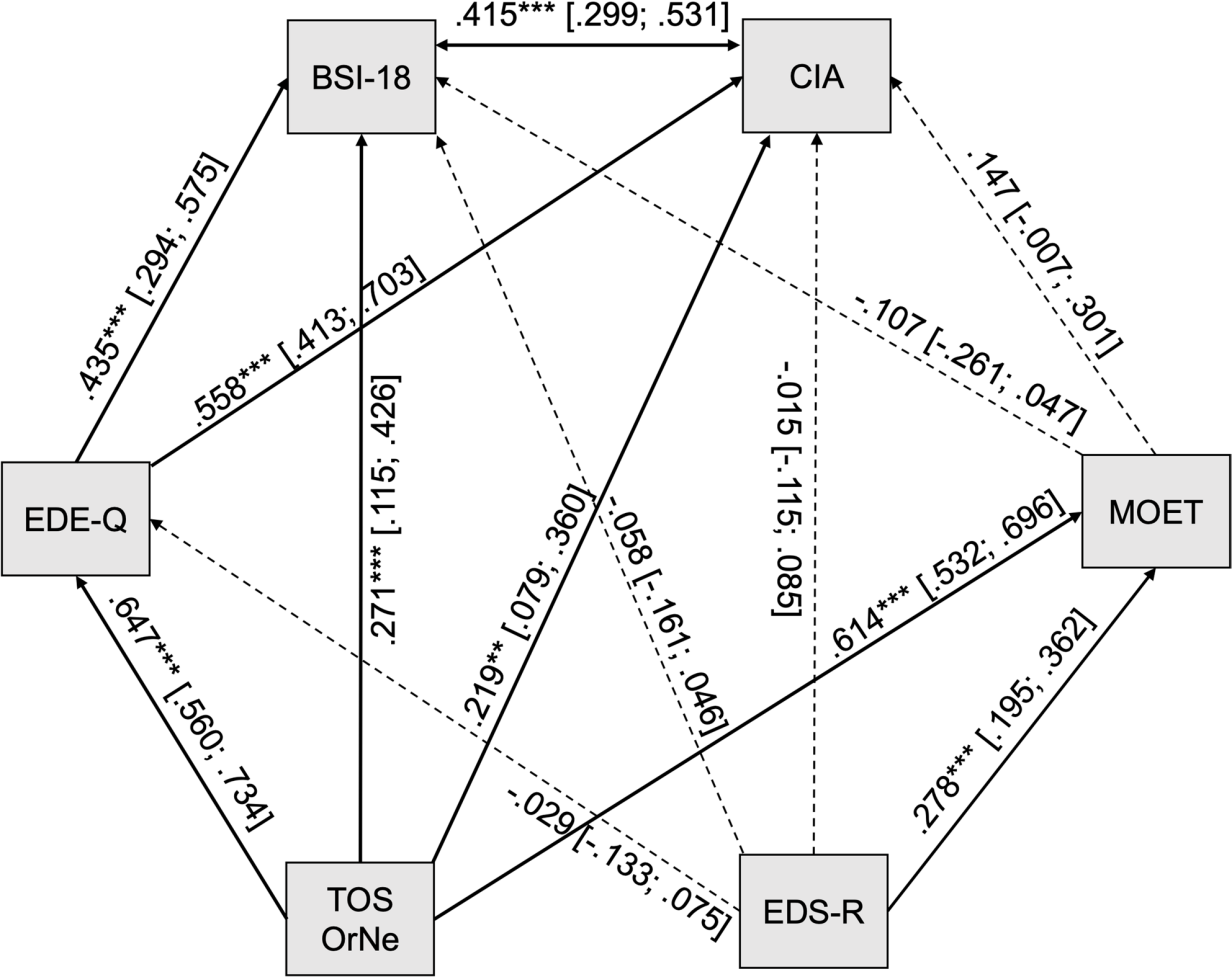
Path analysis with standardized path coefficients, with non-significant paths displayed as dashed. BSI-18: Global Severity Index of the Brief Symptom Inventory 18; CIA: Clinical Impairment Assessment; EDE-Q: Eating Disorders Examination Questionnaire; MOET: Muscularity-Oriented Eating Test; TOS-OrNe: Orthorexia nervosa subscale of the Teruel Orthorexia Scale; EDS-R: Exercise Dependence Scale. *** *p* < 0.001 ** *p* < 0.01 * *p* < 0.05

Subsequently, we exploratively examined all variables of interest in a path analysis to gain insight into direct and indirect effects of TOS-OrNe and EDS-R on the indicators of clinical relevance (see Fig. 2). Model fit indices indicated an acceptable fit to the data, χ² (1) = 38.359, *p* <.001, CFI = 0.969, SRMR = 0.041. In line with the regression analyses, TOS-OrNe had significant direct effects on both CIA and BSI-18. Additionally, indirect effects of TOS-OrNe on these outcomes were observed, with EDE-Q scores significantly mediating the associations with CIA (β = 0.361, 95%-CI [0.254, 0.468], *p* < .001) and BSI-18 (β = 0.281, 95%-CI [0.187, 0.375], *p* < .001). In contrast, MOET (CIA: β = 0.090, 95%-CI [− 0.004, 0.185], *p* = .062; BSI-18: β = −0.066, 95%-CI [− 0.161, 0.030], *p* = .179) did not mediate these associations. EDS-R, on the other hand, showed neither indirect effects – mediated by EDE-Q (CIA: β = −0.016, 95%-CI [− 0.075, 0.043], *p* = .591; BSI-18: β = −0.013, 95%-CI [− 0.059, 0.034], *p* = .594) or MOET (CIA: β = 0.041, 95%-CI [− 0.004, 0.086], *p* = .075; BSI-18: β = −0.030, 95%-CI [− 0.074, 0.014], *p* = .183) – nor direct effects. While TOS-OrNe significantly predicted both EDE-Q and MOET scores, EDS-R only predicted MOET scores.

### Explorative factor analysis

We explored the factor structure and cross-loadings of the items of TOS-OrNe, EDS-R, EDE-Q, and MOET to gain insight into the quality of their overlap. Although no pairwise polychoric correlations exceeded 0.90, the determinant of the correlation matrix was near-zero, and one eigenvalue approached zero (0.003), indicating multivariate multicollinearity among items. Given these findings, we extracted a six-factor solution, as indicated by Velicer’s MAP-test, and not a seven-factor solution suggested by Horn’s parallel analysis to avoid overfitting. However, the sixth factor accounted for only 1.6% of the variance, with just two Items loading above the cut-off (≥ 0.264). Thus, we subsequently conducted a five-factor solution (see Tables S2-S3 in Supplementary Materials). Given the marginal variance explained by Factor 5 (2.3%), its poor factor score adequacy (*R*² = 0.589, minimum correlation = 0.177), and a mean item complexity of 1.5, a four-factor solution appeared more parsimonious and theoretically appropriate than a five-factor model. In line, measures of factor score adequacy were strong across all four factors and primarily loadings largely aligned with the theoretical construct (see Tables S4-S5 in Supplementary Materials).

Specifically, EDE-Q items loaded clearly on Factor 1 (restrictive disordered eating), and EDS-R items on Factor 2 (Exercise Addiction). Factor 3 appeared to capture orthorexic behaviours, with all TOS-OrNe items loading acceptably. However, item complexity of TOS-OrNe was notable. Two TOS-OrNe items – those assessing guilt and self-punishment as responses to dietary violations – showed primary loadings on the restrictive disordered eating factor. Factor 4 represented muscularity-oriented disordered eating, with most MOET items – except for Items 3, 4, and 14 – loading sufficiently. However, eight of the MOET items showed a high complexity or primarily loaded onto the orthorexic eating factor. Overall, cross-loadings further suggested overlap of OrNe with restrictive and muscularity-oriented disordered eating, particularly in terms of cognitive preoccupation, shame or social impairment, and behavioural rigidity. This included impaired concentration due to cognitive preoccupation (EDE-Q Item 7), worry about being observed while eating (EDE-Q Item 21) or others know one’s dietary rules (MOET Item 13), bringing one’s food to social events (MOET Item 6), feeling misunderstood in one’s commitment to dietary rules (MOET Item 14), and prioritizing dietary ideals over work-related obligations (MOET Item 15). Thus, the EDS-R showed a better discriminant validity than TOS-OrNe on item level. On factor level, factor-intercorrelations suggested a strong proximity of orthorexic behaviours with both restrictive (*r* = 0.538) and muscularity-oriented disordered eating (*r* = 0.539). Exercise Addiction showed a strong link with muscularity-oriented disordered eating (*r* = 0.539) as well, but was more closely related to orthorexic behaviours (*r* = 0.486) than restrictive disordered eating (*r* = 0.232).

## Discussion

The study examined the overlap of Orthorexia Nervosa and Exercise Addiction with restrictive and muscularity-oriented disordered eating, and their unique associations with psychological distress and psychosocial impairment in a community sample. We expected gender to moderate these overlaps – specifically, that muscularity-oriented eating behaviours would show stronger associations with EA and OrNe in men, and restrictive disordered eating in women. Partly in line with our hypotheses, we observed gender-specific differences in the correlational patterns of OrNe, but not EA. Exercise Addiction was consistently more closely related to muscularity-oriented than restrictive disordered eating across genders. Orthorexic eating behaviours, on the other hand, showed stronger associations with both restrictive and muscularity-oriented eating pathology among women, contradicting the idea that muscularity may play a higher role in the overlaps among men. Our findings point to gender-specific extents of nosological overlap of OrNe within the spectrum of disordered eating, while gender appeared to play a limited role in the manifestation of EA in the present sample. With regard to clinical indicators, OrNe showed small but statistically significant unique associations with psychological distress and impairment beyond other forms of disordered eating. These effects were both direct and indirect, with restrictive disordered eating acting as a significant mediator in the exploratively path analysis. In contrast, EA did not uniquely predict distress or impairment once shared variance with disordered eating symptoms was accounted for. Overall, OrNe appears to contribute to explain variance in indicators of clinical relevance, whereas EA may primarily reflect underlying muscularity-oriented eating pathology and lacks unique predictive value.

The extremely high correlation between OrNe and muscularity-oriented disordered eating among women (> 0.80) raised concerns regarding their discriminant validity, suggesting a strong conceptual overlap or even redundancy [83]. Latent correlations indicated that orthorexic eating behaviours are closely related to, yet distinct from, both restrictive and muscularity-oriented disordered eating. However, there were considerable cross-loadings on TOS-OrNe Item level with restrictive and muscularity-oriented symptoms – especially regarding the emotional consequences of violating dietary rules and the social centrality of one’s eating practices. In contrast, EDS-R Items formed a more distinct factor, which was also strongly correlated with muscularity-oriented eating behaviours and OrNe, but only slightly with restrictive disordered eating. Accordingly, there was also overlap at the latent level between those constructs.

These findings contribute to the ongoing debate regarding the nosological distinctiveness of OrNe and EA from disordered eating by highlighting the role of muscularity-oriented eating pathology – an aspect that has received limited empirical attention in this context. Both thinness- and muscularity-related motives may play a more central role in orthorexic behaviours than previously assumed, especially among women. Although OrNe accounted for unique variance in clinical indicators in our data, the substantial overlap with other disordered eating patterns challenges the original conceptualization of OrNe as a distinct behavioural pattern revolving only around health [84, 85]. Indeed, network analyses demonstrated the complex interplay between rumination about healthy eating, muscularity concerns, fear and guilt related to weight gain [40, 50]. Various forms of disordered eating – orthorexic, restrictive, and muscularity-disordered eating – may reflect responses to body ideals such as the athletic-feminine ideal socially promoted in the context of healthism and self-optimization [44, 45, 86]. Rather than being distinct, these behaviours could represent multidimensional manifestations of disordered eating shaped by contemporary and varying sociocultural body ideals [87–89].

In the case of EA, its strong association with muscularity-oriented disordered eating behaviours across genders – in the absence of unique links to indicators of clinical relevance – suggests that concerns about muscularity may be closely tied to this behavioural pattern as well. Indeed, EA was previously correlated with body image concerns to a moderate extent [90], even among fitness centre users without signs of disordered eating [91]. Furthermore, EA seems to be more strongly associated with exercise activity in fitness and resistance sports than endurance sports [92] and strongly correlated with symptoms of muscle dysmorphia in general [93]. While most prior studies did not control for disordered eating when examining clinical impairment or distress [11, 17], one study that controlled for eating disorder risk as a binary variable did find a link between Exercise Addiction and psychological distress [7]. This association was not present when we dimensionally controlled for disordered eating. Moreover, existing evidence suggests that EA predominantly occurs alongside eating disorders, thus reflecting instrumental exercise [28, 29]. Taken together, these findings suggest that what is captured as EA largely reflects exercise behaviours embedded in disordered eating, whereas genuine manifestations, if present, appear to be rare in community samples. Importantly, levels of disordered eating were relatively elevated in our sample: 18.8% of participants scored above the EDE-Q cut-off, compared with 3.9% in a large community-based German sample [94]. This context may have decreased the likelihood of detecting true EA in our sample.

Furthermore, a recent mixed-methods study points to false positive results of the EA risk classification based on EDS-R scores by additionally implementing a diagnostic interview. The responses of participants further suggested that item interpretations (e.g. increasement of exercise to achieve desired effects/benefits) may depend on the individual athletic and situational context. Committed athletes could positively respond to such as items even in absence of obsessive-compulsive tendencies [95]. Our findings support the possibility that elevated EDS-R scores may reflect normative trade-offs associated with dedicated athletic commitment or passion rather than clinically relevant life interference [96]. Altogether, further qualitative studies are needed to clarify the extent of distress and clinically significant impairment, while differentially ruling out body image disturbances and disordered eating – particularly those related to muscularity. Existing evidence may in fact be confounded by instrumental exercise [32].

However, the present study has its own limitations. First, due to the cross-sectional design, no causal inferences can be drawn, which limits conclusions regarding etiological pathways. Consequently, it remains unclear whether OrNe and EA tendencies emerge prior to, concurrently with, or as a consequence of disordered eating. Similarly, the role of psychological distress and impairment is uncertain, as they could act as comorbidities, risk factors, or consequences. Second, the BSI-18 covers key symptom domains (somatization, depression, and anxiety), but it does not capture potential distress that may exist outside of these areas. As a result, overall distress associated with OrNe or EA could be underestimated. The CIA is designed to assess psychosocial impairment rather than symptom severity and is suitable for evaluating the incremental contributions of TOS-OrNe and EDS-R beyond the EDE-Q and MOET. However, some shared item content could have slightly inflated the associations, primarily for TOS-OrNe, as no significant incremental associations were observed for EDS-R. Third, the sample consisted primarily of women. This imbalance may have constrained the detection of gender differences in overlap patterns, particularly for EA, where associations with disordered eating behaviours were descriptively stronger among men. Fourth, although we recently translated and validated the newly developed MOET into German, our validation has not yet been published. Moreover, the MOET and TOS-OrNe showed some psychometric limitations within this sample. The exploratory factor analytic results showed that several items displayed high complexity or cross-loadings onto other factors, and factor intercorrelations indicated only partial discriminant validity between constructs. This suggests caution in interpreting the measures’ ability to capture distinct constructs, as observed overlap may reflect limitations of the instruments or the constructs themselves. These limitations highlight the need for future research to refine and validate assessment tools for orthorexic and muscularity-oriented disordered eating, and to clarify the distinctiveness of these constructs. Mixed-methods approaches may further enhance the specificity and contextual validity of screening instruments for OrNe and EA.

## Conclusion

While OrNe’s and EA’s overlap with disordered eating have been increasingly investigated since their conceptual introduction [97, 98], research has overlooked the role of muscularity-oriented behaviours and motives in their development and clinical presentation. Orthorexic behaviours showed unique associations with psychological distress and impairment, but their overlap with restrictive and muscularity-oriented disordered eating indicates that distinguishing them as a separate condition may be challenging. A strong preoccupation with healthy or clean eating could reflect either an aspect of disordered eating itself [85] or a dysfunctional strategy to cope with it while maintaining a sense of control [99]. Correlated with muscularity-oriented disordered eating as well, the conceptualization of EA as a behavioural addiction may have limited validity, given the lack of unique associations with clinical indicators once shared variance is considered [100]. Assuming that ON and EA at least partly reflect aspects of disordered eating, eating disorder research and practice could also consider not only overt dietary restriction or weight-loss motives, but also rigid health- and muscularity-oriented rules [101]. Overall, ongoing debates regarding the nosological status of OrNe and EA could reflect variability in how eating pathology presents across contexts. Given the frequent crossover between established eating disorder categories, it may be helpful to view disordered eating from a multidimensional and integrative perspective [102, 103].

## Supplementary Information

Supplementary Material 1.

## Data Availability

The datasets analyzed during the current study are available from the corresponding author on request.
