# Supplementary material for: Orthorexia nervosa and exercise addiction: distinct entities beyond restrictive and muscularity-oriented disordered eating behaviours?
Source: J Eat Disord. 2026 Jan 25;14:34. doi: 10.1186/s40337-026-01535-8 (PMC12849567; doi:10.1186/s40337-026-01535-8)
Supplement: Supplementary file 1 — Supplementary Material 1. [file 40337_2026_1535_MOESM1_ESM.pdf]

Supplementary Material

Table S1.
Pearson correlation matrix of variables of interest for the total sample (*N* = 384) in the lower triangular section and for women (*N* = 293) / men (*N* = 91) in the higher triangular section.

	TOS-OrNe	EDS-R	EDE-Q	MOET	BSI-18	CIA
TOS-OrNe	-	.430*** / .482***	.676*** / .431*** [a]	.810*** / .524*** [a]	.510*** / .083	.696*** / .522***
EDS-R	.419***	-	.272*** / .334** [b]	.504*** / .604*** [b]	.141* / .079	.276*** / .479***
EDE-Q	.635***	.242***	-	.654*** / .591***	.551*** / .370**	.768*** / .786***
MOET	.730***	.536***	.609***	-	.378*** / .212*	.647*** / .676***
BSI-18	.448***	.105*	.532***	.327***	-	.677*** / .394***
CIA	.667***	.288***	.774***	.632***	.637***	-

Note. CIA = Clinical Impairment Assessment, EDE-Q = Eating Disorders Examination Questionnaire, EDS-R = Exercise Dependence Scale, BSI-18 = Global Severity Index of the Brief Symptom Inventory 18, MOET = Muscularity-Oriented Eating Test, TOS-OrNe = Orthorexia nervosa subscale of the Teruel Orthorexia Scale.[a] Significant gender effect, [b] Not significant gender effect. *** *p* <.001 ** *p* <.01 * *p* <.05.

Table S2.
Factor loadings, communality h^2, uniqueness u^2, and complexity of the items of TOS-OrNe, EDS-R, EDE-Q, and MOET in a five-factor factor solution.

Items	Loadings					h^2	u^2	com
	F 1	F 2	F 3	F 4	F 5			
TOS 4	**.406**	-.057	**.409**	.027	.202	.642	.358	2.50
TOS 5	-.055	.068	**.704**	.169	-.080	.634	.366	1.18
TOS 9	.084	-.016	**.525**	.303	.086	.651	.349	1.73
TOS 10	.215	-.075	**.576**	.032	.140	.571	.429	1.45
TOS 12	.312	.010	**.515**	-.008	.150	.625	.375	1.85
TOS 14	-.139	.091	**.619**	.056	.082	.429	.571	1.20
TOS 16	**.489**	-.056	.367	.072	.099	.668	.332	2.04
TOS 17	.119	.159	**.612**	.167	-.026	.755	.245	1.38
EDS-R 1	-.059	**.437**	.110	-.126	**.433**	.416	.584	2.33
EDS-R 2	.210	**.631**	.006	-.146	.076	.423	.577	1.37
EDS-R 3	-.047	**.675**	-.285	.219	.274	.656	.344	1.97
EDS-R 4	.012	**.666**	.018	.151	.017	.595	.405	1.11
EDS-R 5	-.043	**.604**	.098	.115	-.056	.490	.510	1.16
EDS-R 6	-.121	**.810**	-.165	.111	.128	.690	.310	1.22
EDS-R 7	-.016	**.762**	.065	.053	-.027	.662	.338	1.03
EDS-R 8	.035	.343	.358	-.096	.276	.460	.540	3.06
EDS-R 9	.131	**.693**	.029	-.165	.066	.463	.537	1.21

EDS-R 10	-.030	**.677**	-.179	.123	.265	.606	.394	1.54
EDS-R 11	.070	**.771**	.042	.062	.020	.722	.278	1.04
EDS-R 12	-.077	**.674**	.207	-.015	.040	.600	.400	1.22
EDS-R 13	-.085	**.807**	-.096	.002	.139	.639	.361	1.11
EDS-R 14	-.072	**.769**	.091	.010	.004	.654	.346	1.05
EDS-R 15	-.045	**.513**	.150	-.162	**.455**	.529	.471	2.40
EDS-R 16	.231	**.723**	.065	-.168	-.002	.555	.445	1.34
EDS-R 17	.004	**.737**	-.223	.081	.245	.628	.372	1.44
EDS-R 18	-.015	**.752**	.081	.120	-.059	.712	.288	1.09
EDS-R 19	.039	**.632**	.195	-.018	-.113	.524	.476	1.27
EDS-R 20	-.095	**.797**	-.104	.061	.110	.649	.351	1.11
EDS-R 21	-.008	**.742**	.114	.011	-.015	.644	.356	1.05
EDE-Q 1	**.621**	-.023	-.149	**.401**	.007	.629	.371	1.85
EDE-Q 2	**.592**	.011	.070	.131	-.094	.454	.546	1.18
EDE-Q 3	**.642**	.039	-.148	.252	.069	.570	.430	1.46
EDE-Q 4	**.480**	-.123	-.148	**.616**	-.039	.636	.364	2.13
EDE-Q 5	**.627**	.045	.080	.141	-.044	.560	.440	1.16
EDE-Q 6	**.741**	.131	-.090	.029	.033	.568	.432	1.10
EDE-Q 7	**.463**	-.004	.359	.164	-.113	.619	.381	2.31
EDE-Q 8	**.689**	.068	.084	.092	-.129	.579	.421	1.16
EDE-Q 9	**.746**	-.004	.148	.080	-.015	.759	.241	1.10
EDE-Q 10	**.900**	.054	-.061	-.042	.021	.753	.247	1.02
EDE-Q 11	**.962**	.025	-.204	-.002	-.054	.723	.277	1.10
EDE-Q 12	**.980**	.018	-.278	.070	-.038	.767	.233	1.17
EDE-Q 19	**.553**	-.034	.291	-.171	-.042	.432	.568	1.76
EDE-Q 20	**.798**	.024	-.028	-.061	.115	.659	.341	1.06
EDE-Q 21	**.627**	-.009	.291	-.037	-.166	.573	.427	1.58
EDE-Q 22	**.797**	-.046	.054	-.011	.030	.684	.316	1.02
EDE-Q 23	**.726**	-.090	.133	-.030	.084	.657	.343	1.13
EDE-Q 24	**.585**	.002	.276	-.277	.067	.468	.532	1.94
EDE-Q 25	**.967**	.000	-.208	-.058	-.085	.679	.321	1.12
EDE-Q 26	**.964**	-.009	-.177	-.100	-.022	.702	.298	1.09
EDE-Q 27	**.923**	-.009	-.078	-.109	-.044	.684	.316	1.05
EDE-Q 28	**.865**	-.061	-.012	-.072	-.076	.639	.361	1.04
MOET 1	.058	.016	-.033	**.774**	-.067	.584	.416	1.03
MOET 2	.081	.149	.146	**.553**	-.113	.539	.461	1.43
MOET 3	.272	-.065	.340	.144	.274	.559	.441	3.37
MOET 4	.154	-.114	**.444**	.208	.224	.544	.456	2.43
MOET 5	.119	.093	.263	**.544**	.021	.721	.279	1.63
MOET 6	-.229	-.115	**.533**	**.519**	-.167	.517	.483	2.67
MOET 7	.387	.032	.100	**.443**	.017	.636	.364	2.09
MOET 8	-.053	-.012	.167	**.609**	.015	.469	.531	1.17
MOET 9	-.144	.280	.148	**.450**	-.093	.428	.572	2.29
MOET 10	-.090	.152	.168	**.663**	-.117	.605	.395	1.35

MOET 11	.074	.198	.073	**.556**	.057	.614	.386	1.35
MOET 12	.262	-.036	.231	**.420**	.149	.658	.342	2.64
MOET 13	.227	-.037	**.499**	.272	.005	.670	.330	2.01
MOET 14	-.007	.017	**.547**	.303	.069	.609	.391	1.60
MOET 15	-.140	.027	**.485**	**.458**	.029	.604	.396	2.18

Note. Practically useful factor loadings ≥ .264 are highlighted in grey, and factor loadings factor loadings ≥ .400 are presented in bold.

Table S3.
Factor intercorrelations and measures of factor adequacy of the five-factor solution of the exploratory factor analysis.

	F 1	F 2	F 3	F 4	F5
Intercorrelations					
F2	.168	-			
F3	.541	.465	-		
F4	.456	.500	.523	-	
F5	.392	.231	.194	.429	-
Multiple R^2 of scores with factors	.944	.917	.859	.853	.589
Minimum correlation of possible factor scores	.887	.835	.717	.706	.177

Table S4.
Factor loadings, communality h^2, uniqueness u^2, and complexity of the items of TOS-OrNe, EDS-R, EDE-Q, and MOET in a four-factor factor solution (mean item complexity of 1.4).

	Loadings						
Items	Factor 1 (restr. dis. eating)	Factor 2 (Exercise Addiction)	Factor 3 (orthorexic behaviours)	Factor 4 (musc. dis. eating)	h2	u2	Com
TOS 4	**.498**	-.005	.348	.071	.616	.384	1.84
TOS 5	-.076	.042	**.730**	.136	.629	.371	1.10
TOS 9	.130	.005	**.504**	.314	.646	.354	1.83
TOS 10	.286	-.041	**.534**	.061	.557	.443	1.58
TOS 12	.380	.048	**.472**	.021	.612	.388	1.94
TOS 14	-.102	.112	**.602**	.064	.425	.575	1.15
TOS 16	**.541**	-.033	.334	.091	.662	.338	1.74
TOS 17	.111	.149	**.626**	.145	.753	.247	1.30
EDS-R 1	.080	**.559**	.009	-.038	.322	.678	1.05
EDS-R 2	.198	**.655**	.007	-.149	.423	.577	1.29
EDS-R 3	.010	**.758**	-.329	.261	.635	.365	1.63
EDS-R 4	-.024	**.675**	.042	.129	.590	.410	1.08
EDS-R 5	-.102	**.592**	.139	.077	.476	.524	1.21
EDS-R 6	-.127	**.853**	-.165	.113	.690	.310	1.16
EDS-R 7	-.075	**.758**	.103	.017	.648	.352	1.06
EDS-R 8	.125	**.419**	.296	-.046	.426	.574	2.04
EDS-R 9	.111	**.714**	.036	-.173	.462	.538	1.17
EDS-R 10	.025	**.756**	-.223	.163	.586	.414	1.27
EDS-R 11	.029	**.781**	.068	.037	.715	.285	1.02
EDS-R 12	-.102	**.688**	.223	-.033	.598	.402	1.26

EDS-R 13	-.086	**.851**	-.101	.007	.639	.361	1.05
EDS-R 14	-.120	**.774**	.121	-.019	.646	.354	1.10
EDS-R 15	.097	**.642**	.045	-.072	.430	.570	1.08
EDS-R 16	.184	**.725**	.090	-.195	.546	.454	1.31
EDS-R 17	.046	**.811**	-.259	.115	.615	.385	1.25
EDS-R 18	-.085	**.740**	.128	.075	.692	.308	1.11
EDS-R 19	-.042	**.602**	.250	-.071	.494	.506	1.38
EDS-R 20	-.107	**.833**	-.100	.059	.649	.351	1.07
EDS-R 21	-.060	**.742**	.148	-.021	.633	.367	1.09
EDE-Q 1	**.629**	-.020	-.152	**.400**	.629	.371	1.84
EDE-Q 2	**.561**	-.017	.092	.104	.445	.555	1.13
EDE-Q 3	**.669**	.058	-.168	.265	.568	.432	1.47
EDE-Q 4	**.477**	-.133	-.139	**.605**	.635	.365	2.13
EDE-Q 5	**.613**	.030	.090	.125	.556	.444	1.13
EDE-Q 6	**.748**	.139	-.100	.032	.568	.432	1.11
EDE-Q 7	**.430**	-.038	.387	.130	.608	.392	2.19
EDE-Q 8	**.641**	.030	.116	.055	.562	.438	1.09
EDE-Q 9	**.748**	-.011	.147	.072	.758	.242	1.10
EDE-Q 10	**.908**	.057	-.073	-.039	.753	.247	1.02
EDE-Q 11	**.942**	.008	-.197	-.015	.718	.282	1.09
EDE-Q 12	**.965**	.006	-.275	.062	.764	.236	1.17
EDE-Q 19	**.546**	-.050	.294	-.183	.430	.570	1.81
EDE-Q 20	**.845**	.054	-.066	-.035	.653	.347	1.02
EDE-Q 21	**.572**	-.060	.330	-.080	.550	.450	1.67
EDE-Q 22	**.817**	-.041	.037	-.004	.683	.317	1.01
EDE-Q 23	**.770**	-.070	.100	-.009	.653	.347	1.05
EDE-Q 24	**.617**	.016	.249	-.262	.467	.533	1.71
EDE-Q 25	**.935**	-.026	-.194	-.076	.670	.330	1.10
EDE-Q 26	**.957**	-.017	-.181	-.103	.701	.299	1.10
EDE-Q 27	**.910**	-.025	-.075	-.119	.681	.319	1.05
EDE-Q 28	**.844**	-.085	-.002	-.088	.633	.367	1.04
MOET 1	.036	.000	-.006	**.748**	.581	.419	1.00
MOET 2	.035	.120	.188	**.512**	.526	.474	1.39
MOET 3	.390	.008	.262	.206	.507	.493	2.34
MOET 4	.258	-.055	.380	.257	.506	.494	2.64
MOET 5	.130	.099	.266	**.535**	.721	.279	1.69
MOET 6	-.274	-.163	**.581**	**.470**	.503	.497	2.58
MOET 7	.399	.037	.098	**.439**	.636	.364	2.10
MOET 8	-.039	-.006	.170	**.604**	.469	.531	1.17
MOET 9	-.192	.257	.191	**.410**	.416	.584	2.68
MOET 10	-.137	.122	.214	**.620**	.592	.408	1.43

MOET 11	.089	.216	.071	**.554**	.613	.387	1.39
MOET 12	.331	.004	.192	**.449**	.643	.357	2.24
MOET 13	.244	-.039	**.498**	.265	.670	.330	2.06
MOET 14	.030	.034	**.533**	.308	.606	.394	1.62
MOET 15	-.119	.034	**.484**	**.453**	.603	.397	2.13

Note. Practically useful factor loadings ≥ .264 are highlighted in grey, and factor loadings factor loadings ≥ .400 are presented in bold.

Table S5.

Factor intercorrelations and measures of factor adequacy of the four-factor solution (overall variance explanation = 59.2%) of the exploratory factor analysis.

	F 1 (restr. dis. eating)	F 2 (Exercise Addiction)	F 3 (orthorexic behaviours)	F 4 (musc. dis. eating)
Intercorrelations				
F2 (Exercise Addiction)	.232	-		
F3 (orthorexic behaviours)	.538	.486	-	
F4 (musc. dis. eating)	.452	.539	.520	-
Multiple R^2 of scores with factors	.945	.928	.859	.849
Minimum correlation of possible factor scores	.889	.855	.719	.698
